# Exploring soliton solutions and dynamical features of three dimensional Gardner Kadomtsov Petviashvili equation

**DOI:** 10.1038/s41598-025-22968-2

**Published:** 2025-10-23

**Authors:** Amjad Hussain, Muhammad Zeeshan, Muhammad Junaid U Rehman, Adil Jhangeer

**Affiliations:** 1 Department of Mathematics, Quaid-i-Azam University, 45320, Islamabad, 44000 Pakistan; 2 Department of Automation, Biomechanics, and Mechatronics, Lodz University of Technology, 1/15 Stefanowski st., 90- 537, Łódź, Poland; 3 IT4Innovations, VSB-Technical University of Ostrava, Ostrava-Poruba, Czech Republic; 4 Center for Theoretical Physics, Khazar University, 41 Mehseti Str., Baku, AZ1096 Azerbaijan

**Keywords:** (3+1)-dimensional Gardner–Kadomtsov–Petviashvili equation, Soliton solutions, Jacobi elliptic function method, Phase portraits, Quasi-periodic and chaotic behavior, Mathematics and computing, Physics

## Abstract

In this paper, the dynamical features and soliton structures of the Gardner-Kadomtsov-Petviashvili equation in three dimensions are looked at. The Jacobi elliptic function method yields wave solutions that display distinct behaviors based on parameter variations. We reformulate the system into a planar dynamical system via the Galilean transformation for further analysis.Phase portraits are depicted by adjusting the bifurcation parameters , while periodic and super nonlinear periodic wave solutions are portrayed using numerical simulations. Furthermore, quasi-periodic and chaotic behavior is depicted by varying the external forcing term and using tools such as Lyapunov exponents, Poincaré maps, and sensitivity analysis. Changes in frequency and amplitude strongly influence the system’s dynamics, offering insights that can improve predictions, enhance control methods, and optimize model performance.

## Introduction

The investigation of the dynamical features of partial differential equations (PDEs) via different dynamics tools, such as bifurcation theory and sensitivity analysis, is ongoing. Such investigations play a prime role in the study of complex phenomena in optical systems, quantum physics, and fluid dynamics. Although considerable progress has been achieved, chaos in complex systems is still far from fully comprehended by any researcher^1^. The detection of chaos in nonlinear systems requires appropriate analytical techniques with phase portraits, bifurcation diagrams, power spectrum analysis, nonlinear time series analysis, Poincaré maps, and Lyapunov exponents, some of which may provide appropriate insight into the particular situation of the systems under study. Due to this complexity, a strong analysis will often adopt multiple methodologies, ensuring an in-depth understanding of this system^2,3^. Recently, several dynamic properties of different PDEs have attracted much attention because most of these equations admit localized solutions called solitons, among other forms of traveling waves. In this respect, the analysis of bifurcation and chaos theory is complementary, since the former investigates how changes in parameters affect stability and spatial patterns, while the latter points out the thresholds beyond which instability and chaotic behavior arise^4,5^. Together, these methods shed light on the interplay between stability and chaos^6–8^, which is important to understand the underlying dynamics of nonlinear systems. Moreover, related non-linear equations are often studied using bifurcation analysis and chaos detection methods^17–19^. For example, the bifurcation and chaotic behaviors of the KdV-MKdV equations^9^ have been studied in detail and provide important information about complex wave phenomena. However, the study of soliton solutions to non-linear systems is equally important, as it has many applications in science and engineering. Soliton solutions help us to understand the complex behavior of nonlinear systems. The significance of soliton solutions in several physical contexts, such as wave propagation and optical systems, has been highlighted in earlier research^10–14^.

The Gardner equation models internal waves in stratified fluids and has applications in plasma physics^15^ and Bose-Einstein condensates^16^. The multi-dimensional Gardner-KP equation is vital in ocean engineering, describing nonlinear internal waves on ocean shelves. Studies have shown its role in governing dispersive surface waves near critical depths^20^, dispersive shock waves through the cylindrical Gardner equation^21^, and solitary wave solutions^22^. In addition, elliptic and traveling wave solutions^23^ and solitons have been developed using the Hirota bilinear method^24^. The (3 + 1)-dimensional Gardner-KP equation is an extended form of the (2 + 1)-dimensional Gardner-KP equation^25,26^, which serves as the primary focus of this study.

The (3+1)-dimensional nonlinear Gardner–Kadomtsov–Petviashvili (Gardner-KP) equation, a notable nonlinear partial differential equation, serves as an important model for examining soliton dynamics and chaotic phenomena:

1 $$\begin{aligned} \left( \Psi _\sigma + 6\Psi \Psi _\phi - 6 \Psi ^2 \Psi _\phi + \nu ^2 \Psi _{\phi \phi \phi } \right) _\phi + \mu \left( \Psi _{\beta \beta } + \Psi _{\xi \xi }\right) = 0,\end{aligned}$$

where $\mu =\pm 1$ and $\Psi = \Psi (\phi , \beta , \xi , \sigma )$ is the real field representing the amplitude of the wave, $\sigma$ is the temporal component, and $\phi$, $\beta$, $\xi$ are the spatial components.

Equation (1) models the evolution of the nonlinear wave amplitude $\Psi (\phi , \beta , \xi , \sigma )$ in a multidimensional medium. The terms appearing in (1) have the following physical meanings:

- $\Psi _\sigma$: temporal change of the wave amplitude.
- $6 \Psi \Psi _\phi$: quadratic nonlinearity representing wave steepening effects.
- $-6 \Psi ^2 \Psi _\phi$: cubic nonlinearity accounting for higher-order nonlinear interactions.
- The term $\nu ^2 \Psi _{\phi \phi \phi }$ is used for dispersive behavior caused by waves spreading and dispersing in the $\phi$ direction and $\nu ^2$ controls how much dispersion there is.
- $\mu (\Psi _{\beta \beta } + \Psi _{\xi \xi })$, that describes wave modulation in $\beta$ and $\xi$ directions and $\mu = \pm 1$ shows if this is dispersion or anti-dispersion.

Despite the numerous existing works on lower-dimensional Gardner-KP equations, they seem to lack comprehensive coverage of the (3+1)-dimensional case, particularly in terms of its explicit soliton solutions and intricate soliton dynamics. It is complicated to study the influence of various features in systems that are nonlinear, and such analysis usually requires sophisticated methods to show chaos and supernonlinear waves. We address these gaps by employing the Jacobi elliptic function method to derive new exact solutions and by investigating various aspects of the nonlinear model, as it is significant for ocean engineering, plasma physics, and wave studies.

Consequently, this paper aims to derive soliton solutions by the JEF technique^27^ and to analyze the dynamics^28^ of the (3 + 1)-dimensional Gardner-KP equation. This research is centred on achieving two key objectives The first one is to derive exact soliton solutions of the (3+1)-dimensional nonlinear Gardner–Kadomtsov–Petviashvili (Gardner-KP) equation using the efficient JEF technique, and the second is to investigate its nonlinear dynamics through advanced analytical tools, including chaos detection methods, Lyapunov exponents, Poincaré maps, sensitivity analysis, and phase plane analysis. These methods offer a systematic framework for investigating the equation’s shift from regular behavior to chaotic dynamics.

By using a thorough methodology to reveal the soliton dynamics and nonlinear features of the (3+1)-dimensional nonlinear Gardner–Kadomtsov–Petviashvili (Gardner-KP), this study expands and improves upon previous research. By examining the chaotic and bifurcation aspects of the system, this work fills in knowledge gaps in the study of solitons and opens the door to more extensive applications in science and engineering.

Findings from this problem are very different from results obtained in earlier studies based on lower-dimensional Gardner-KP equations^24^. Unlike previous works, we highlight new and complex dynamical behaviors and additional, richer soliton structures of the (3+1)-dimensional model. The soliton solutions found using the Jacobi elliptic function method reveal new ways that pulses can interact and take shape, which are more advanced than what was seen in traditional results. Stability tests show that the results remain reliable even when changing parameters, and further analysis using phase portraits and Lyapunov exponents reveals complex stability patterns, different levels of chaos and additional features not seen in lower dimensions. This comparison shows how significant the effects of multidimensional nonlinear wave propagation are and highlights the main Gardner-KP equations used in this research, which help both the theory and practical applications.

The structure of this paper is as follows: The first part uses the Jacobi elliptic method to find the soliton solutions of the (3+1)-dimensional Gardner-KP equation. In the second part, the dynamic behaviour of the same model is analyzed using various tools, such as phase portraits, bifurcation analysis, and Lyapunov exponents, to investigate its chaotic and quasiperiodic dynamics.

## Soliton Solution of the (3 + 1)-dimensional Gardner-KP equation by JEF Technique

Jacobi elliptic functions, first described by Carl Gustav Jacob Jacobi in 1827, are periodic functions derived as the inverses of elliptic integrals. They are essential in areas such as mathematical physics, nonlinear dynamics and differential equations. The main Jacobi elliptic functions are $\textrm{sn}(z|m)$, $\textrm{cn}(z|m)$, and $\textrm{dn}(z|m)$. They are defined in terms of a parameter *m*, which is called the elliptic modulus, and they show how the amplitude of the elliptic integral is related to its arguments. Because they are periodic and relate to each other, they are like trigonometric functions in elliptic geometry. This gives us many ways to solve hard math and science problems^29^. The detailed description of the method is provided in^27^.

We use the following transformation:

2 $$\begin{aligned} \Psi (\phi , \beta , \xi , \sigma ) = \Upsilon (\chi ), \quad \text {where} \quad \chi = b_1 \phi + b_2 \beta + b_3 \xi + c \sigma , \end{aligned}$$

which defines a traveling wave solution, where

- $b_1, b_2, b_3$ are wave numbers corresponding to spatial directions $\phi$, $\beta$, and $\xi$, representing the direction and wavelength of the wave.
- $c$ is the wave speed in time, governing how fast the wave profile moves.

This transformation reduces the partial differential equation (1) to an ordinary differential equation (ODE) in $\chi$:

3 $$\begin{aligned} (\mu b_3^2 + c b_1 + \mu b_2^2 )\Upsilon '' + 6 b_1^2 (\Upsilon \Upsilon ')' - 6 b_1^2(2\Upsilon (\Upsilon ')^2 + \Upsilon ^2 \Upsilon '') + \nu ^2 b_1^4 \Upsilon '''' = 0, \end{aligned}$$

facilitating the analysis of soliton solutions propagating in multiple spatial dimensions.

Upon the JEF method, we take the solution of Eq. (3) in the following form:

$$\begin{aligned} \Upsilon (\chi ) = \sum _{l=1}^{N} k_l G^l(\chi ). \end{aligned}$$

Next, by virtue of the homogeneous balancing principle, that is balancing the highest-order derivative term with the nonlinear term, we get $N=1$, so the solution simplifies to

4 $$\begin{aligned} \Upsilon (\chi ) = k_0 + k_1 G(\chi ) , \end{aligned}$$

where $k_0$ and $k_1$ are the constants and the function $G(\chi )$ satisfy the followingg ansatz

$$G'(\chi ) = \sqrt{r_1 + r_2 G^2(\chi ) + \frac{r_3}{2} G^4(\chi )}.$$

By substituting (4) into equation (3), we derive a system of equations. We solve this system using the computational software Maple to obtain the following parameter values.

5 $$\begin{aligned} \left\{ \begin{array}{l} \mu = \mu , \quad c = c , \quad v = v, \quad k_0 = \frac{1}{2}, \quad k_1 = k_1, \quad b_1 = b_1, \quad b_2 = b_2 ,\quad b_3 = b_3 , \\ r_1 = r_1, \quad r_2 = -\frac{1}{2} \times \frac{2 \mu b_2^2 + 2 \mu b_3^2 + 3 b_1^2 + 2 \left( {c} \cdot b_1 \right) }{v^2 b_1^2}, \\ r_3 = \frac{2 k_1^2}{v^2}. \end{array} \right. \end{aligned}$$

**Family 1:** When the parameters are defined as $r_1 = 1$, $r_2 = -(1 + \delta ^2)$, and $r_3 = 2 \delta ^2$, the wave profile can be derived from analyzing $G(\chi )$, where the function is expressed using the Jacobi elliptic sine function, $\operatorname {sn}(\chi , \delta )$:

$$\begin{aligned} \Upsilon (\chi ) = k_0 + k_1 \operatorname {sn}(\chi , \delta ). \end{aligned}$$

Specifically, in this case, the wave profile simplifies to

$$\begin{aligned} \Upsilon (\chi ) = \frac{1}{2} + k_1 \operatorname {sn}(\chi , \delta ). \end{aligned}$$

As the parameter $\delta$ approaches 1, the shock wave profile transitions into the following form, represented using the hyperbolic tangent function:

$$\begin{aligned} \Upsilon (\chi ) = \frac{1}{2} + k_1 \tanh (\chi ). \end{aligned}$$

To further generalize this result, we can express the shock wave profile as follows:

6 $$\begin{aligned} \Psi (\phi , \beta , \xi , \sigma ) = \frac{1}{2} + k_1 \tanh (b_1 \phi + b_2 \beta + b_3 \xi + c \sigma ). \end{aligned}$$

**Family 2:** Consider the parameters $r_1 = -\delta ^2(1 - \delta ^2)$, $r_2 = 2\delta ^2 - 1$, and $r_3 = 2$. By choosing $G(\chi ) = \operatorname {ds}(\chi , \delta )$, we derive a periodic wave profile given by:

$$\begin{aligned} \Upsilon (\chi ) = \frac{1}{2} + k_1 \operatorname {ds}(\chi , \delta ). \end{aligned}$$

When the parameter $\delta$ approaches 1, the wave profile transitions into the following form involving the hyperbolic cosecant function:

$$\begin{aligned} \Upsilon (\chi ) = \frac{1}{2} + k_1 \operatorname {csch}(\chi ). \end{aligned}$$

This result can be further generalized for a multidimensional scenario, where the wave profile is expressed as:

$$\begin{aligned} \Psi (\phi , \beta , \xi , \sigma ) = \frac{1}{2} + k_1 \operatorname {csch}(b_1 \phi + b_2 \beta + b_3 \xi + c \sigma ). \end{aligned}$$

**Family 3:** When the parameters are defined as $r_1 = 1 - \delta ^2$, $r_2 = 2 - \delta ^2$, and $r_3 = 2$, the periodic wave profile can be derived by analyzing $G(\chi ) = \operatorname {cs}(\chi , \delta )$. This leads to the expression:

$$\begin{aligned} \Upsilon (\chi ) = \frac{1}{2} + k_1 \operatorname {cs}(\chi , \delta ). \end{aligned}$$

As the parameter $\delta$ approaches the limiting case of $\delta \rightarrow 1$, the profile transforms into:

$$\begin{aligned} \Upsilon (\chi ) = \frac{1}{2} + k_1 \coth (\chi ). \end{aligned}$$

Extending this into a multidimensional framework, the wave profile is given by:

$$\begin{aligned} \Psi (\phi , \beta , \xi , \sigma ) = \frac{1}{2} + k_1 \coth (b_1 \phi + b_2 \beta + b_3 \xi + c \sigma ). \end{aligned}$$

**Family 4:** For the parameters $r_1 = 1 - \delta ^2$, $r_2 = 2\delta ^2 - 1$, and $r_3 = -2\delta ^2$, the periodic wave structure can be described by analyzing $G(\chi ) = \operatorname {cn}(\chi , \delta )$ The resulting wave form is:

$$\begin{aligned} \Upsilon (\chi ) = \frac{1}{2} + k_1 \operatorname {cn}(\chi , \delta ). \end{aligned}$$

In the special case where $\delta \rightarrow 1$, the wave profile transitions to the following form involving the hyperbolic secant function:

$$\begin{aligned} \Upsilon (\chi ) = \frac{1}{2} + k_1 \operatorname {sech}(\chi ). \end{aligned}$$

Moreover, the generalized multidimensional form of the wave profile is expressed as:

7 $$\begin{aligned} \Psi (\phi , \beta , \xi , \sigma ) = \frac{1}{2} + k_1 \operatorname {sech}(b_1 \phi + b_2 \beta + b_3 \xi + c \sigma ). \end{aligned}$$

**Family 5:** For the parameters $r_1 = \delta ^2 - 1$, $r_2 = 2 - \delta ^2$, and $r_3 = -2$, the periodic wave profile can be determined by analyzing $G(\chi ) = \operatorname {dn}(\chi , \delta )$. The expression for the wave profile is given by:

$$\begin{aligned} \Upsilon (\chi ) = \frac{1}{2} + k_1 \operatorname {dn}(\chi , \delta ). \end{aligned}$$

In the limiting case as $\delta \rightarrow 1$, the wave profile simplifies to the hyperbolic secant form:

$$\begin{aligned} \Upsilon (\chi ) = \frac{1}{2} + k_1 \operatorname {sech}(\chi ). \end{aligned}$$

Furthermore, the wave profile can be extended into a multidimensional representation as follows:

$$\begin{aligned} \Psi (\phi , \beta , \xi , \sigma ) = \frac{1}{2} + k_1 \operatorname {sech}(b_1 \phi + b_2 \beta + b_3 \xi + c \sigma ). \end{aligned}$$

**Family 6:** For the parameters $r_1 = \frac{1}{4}$, $r_2 = \frac{\delta ^2 - 2}{2}$, and $r_3 = \frac{\delta ^2}{2}$, the double-periodic wave structure is described by analyzing $G(\chi ) = \frac{\operatorname {sn}(\chi , \delta )}{1 \pm \operatorname {dn}(\chi , \delta )}$. The resulting wave form is:

$$\Upsilon (\chi ) = \frac{1}{2} + \frac{k_1 \operatorname {sn}(\chi , \delta )}{1 \pm \operatorname {dn}(\chi , \delta )}.$$

In the special case where $\delta \rightarrow 1$, the wave profile becomes:

8 $$\begin{aligned} \Psi (\phi , \beta , \xi , \sigma ) = \frac{1}{2} + \frac{k_1 \tanh (\ b_1 \phi + b_2 \beta + b_3 \xi + c \sigma )}{1 \pm \operatorname {sech}(b_1 \phi + b_2 \beta + b_3 \xi + c \sigma )} . \end{aligned}$$

**Family 7:** For the parameters $r_1 = \frac{\delta ^2}{4}$, $r_2 = \frac{\delta ^2 - 2}{2}$, and $r_3 = \frac{\delta ^2}{2}$, the double-periodic wave structure is described by analyzing $G(\chi ) = \frac{\operatorname {sn}(\chi , \delta )}{1 \pm \operatorname {dn}(\chi , \delta )}$. The resulting wave form is:

$$\Upsilon (\chi ) = \frac{1}{2} + \frac{k_1 \operatorname {sn}(\chi , \delta )}{(\delta ^2+1)(\operatorname {sn}(\chi , \delta )1 \pm \operatorname {dn}(\chi , \delta ))}.$$

In the special case where $\delta \rightarrow 1$, the wave profile transitions to:

$$\Upsilon (\chi ) = \frac{1}{2} + \frac{k_1 \tanh (\chi )}{1 \pm \operatorname {sech}(\chi )}.$$

$$\Psi (\phi , \beta , \xi , \sigma ) = \frac{1}{2} + \frac{k_1 \tanh (\ b_1 \phi + b_2 \beta + b_3 \xi + c \sigma )}{1 \pm \operatorname {sech}(\ b_1 \phi + b_2 \beta + b_3 \xi + c \sigma )}.$$

**Family 8:** For the parameters $r_1 = -\frac{(1 - \delta ^2)^2}{4}$, $r_2 = \frac{\delta ^2 + 1}{2}$, and $r_3 = -\frac{1}{2}$, the double-periodic wave structure is described by analyzing $G(\chi ) = \operatorname {cn}(\chi , \delta ) \pm \operatorname {dn}(\chi , \delta )$. The resulting wave form is:

$$\Upsilon (\chi ) = \frac{1}{2} + k_1 (\operatorname {cn}(\chi , \delta ) \pm \operatorname {dn}(\chi , \delta )).$$

In the special case where $\delta \rightarrow 1$, the wave profile becomes:

$$\begin{aligned} \Upsilon (\chi )= & \frac{1}{2} + k_1 (\operatorname {sech}(\chi ) \pm \operatorname {sech}(\chi )).\\ \Psi (\phi , \beta , \xi , \sigma )= & \frac{1}{2} + k_1 (\operatorname {sech}(b_1 \phi + b_2 \beta + b_3 \xi + c \sigma ) \pm \operatorname {sech}(\ b_1 \phi + b_2 \beta + b_3 \xi + c \sigma )). \end{aligned}$$

**Family 9:** For the parameters $r_1 = \frac{\delta ^2 - 1}{4}$, $r_2 = \frac{\delta ^2 + 1}{2}$, and $r_3 = \frac{\delta ^2 - 1}{2}$, the double-periodic wave structure is described by analyzing $G(\chi ) = \frac{\operatorname {dn}(\chi , \delta )}{1 \pm \operatorname {sn}(\chi , \delta )}$. The resulting wave form is:

$$\Upsilon (\chi ) = \frac{1}{2} + \frac{k_1 \operatorname {dn}(\chi , \delta )}{1 \pm \operatorname {sn}(\chi , \delta )}.$$

In the special case where $\delta \rightarrow 1$, the wave profile transitions to:

$$\begin{aligned} \Upsilon (\chi )= & \frac{1}{2} + \frac{k_1 \operatorname {sech}(\chi )}{1 \pm \tanh (\chi )}.\\ \Psi (\phi , \beta , \xi , \sigma )= & \frac{1}{2} + \frac{k_1 \operatorname {sech}(\ b_1 \phi + b_2 \beta + b_3 \xi + c \sigma )}{1 \pm \tanh (\ b_1 \phi + b_2 \beta + b_3 \xi + c \sigma )}. \end{aligned}$$

**Family 10:** For the parameters $r_1 = \frac{1 - \delta ^2}{4}$, $r_2 = \frac{1 - \delta ^2}{2}$, and $r_3 = \frac{1 - \delta ^2}{2}$, the double-periodic wave structure is described by analyzing $G(\chi ) = \frac{\operatorname {cn}(\chi , \delta )}{1 \pm \operatorname {sn}(\chi , \delta )}$. The resulting wave form is:

$$\Upsilon (\chi ) = \frac{1}{2} + \frac{k_1 \operatorname {cn}(\chi , \delta )}{1 \pm \operatorname {sn}(\chi , \delta )}.$$

In the special case where $\delta \rightarrow 1$, the wave profile transitions to:

$$\begin{aligned} \Upsilon (\chi )= & \frac{1}{2} + \frac{k_1 \operatorname {sech}(\chi )}{1 \pm \tanh (\chi )}.\\ \Psi (\phi , \beta , \xi , \sigma )= & \frac{1}{2} + \frac{k_1 \operatorname {sech}(\ b_1 \phi + b_2 \beta + b_3 \xi + c \sigma )}{1 \pm \tanh (\ b_1 \phi + b_2 \beta + b_3 \xi + c \sigma )}. \end{aligned}$$

**Family 11:** For the parameters $r_1 = \frac{1}{4}$, $r_2 = \frac{(1 - \delta ^2)^2}{2}$, and $r_3 = \frac{(1 - \delta ^2)^2}{2}$, the double-periodic wave structure is described by analyzing $G(\chi ) = \frac{\operatorname {sn}(\chi , \delta )}{\operatorname {dn}(\chi , \delta ) \pm \operatorname {cn}(\chi , \delta )}$. The resulting wave form is:

$$\Upsilon (\phi , \beta , \xi , \sigma ) = \frac{1}{2} + \frac{k_1 \operatorname {sn}(\chi , \delta )}{\operatorname {dn}(\chi , \delta ) \pm \operatorname {cn}(\chi , \delta )}.$$

In the special case where $\delta \rightarrow 1$, the wave profile becomes:

$$\Upsilon (\chi ) = \frac{1}{2} + \frac{k_1 \tanh (\chi )}{\operatorname {sech}(\chi ) \pm \operatorname {sech}(\chi )}.$$

9 $$\begin{aligned} \Psi (\phi , \beta , \xi , \sigma ) = \frac{1}{2} + \frac{k_1 \tanh (\ b_1 \phi + b_2 \beta + b_3 \xi + c \sigma )}{\operatorname {sech}(\ b_1 \phi + b_2 \beta + b_3 \xi + c \sigma ) \pm \operatorname {sech}(\ b_1 \phi + b_2 \beta + b_3 \xi + c \sigma )}. \end{aligned}$$

**Family 12:** For the parameters $r_1 = 0$, $r_2 = 0$, and $r_3 = 2$, the rational structure is described by analyzing $G(\chi ) = \frac{D}{\chi }$. The resulting wave form is:

$$\Upsilon (\chi ) = \frac{1}{2} + \frac{k_1 D}{\chi }.$$

In the special case where $\delta \rightarrow 1$, the expression remains:

$$\Psi (\phi , \beta , \xi , \sigma ) = \frac{1}{2} + \frac{k_1 D}{(\ b_1 \phi + b_2 \beta + b_3 \xi + c \sigma )}.$$

**Family 13:** For the parameters $r_1 = 0$, $r_2 = 1$, and $r_3 = 0$, the rational structure is described by analyzing $G(\chi ) = \frac{1}{2} + D e^\chi$. The resulting wave form is:

$$\Upsilon (\chi ) = \frac{1}{2} + b_1 D e^\chi .$$

In the special case where $\delta \rightarrow 1$, the expression remains:

$$\Psi (\phi , \beta , \xi , \sigma ) = \frac{1}{2} + b_1 D e^{(\ b_1 \phi + b_2 \beta + b_3 \xi + c \sigma )}.$$

### Analyzing physical dynamics through visualization

**Fig. 1:**
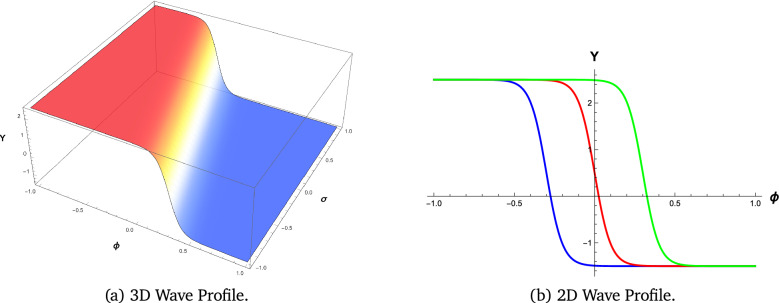
Wave Profile Visualization of the equation (6) with parameters $k_1 = 2$, $b_1 = 10$, $b_2 = 5$, $b_3 = -10$, and $c = 3$.Figures generated using Mathematica Version 13.3.1.0 (Wolfram Research, Champaign, IL, USA; https://www.wolfram.com/mathematica/).

**Fig. 2:**
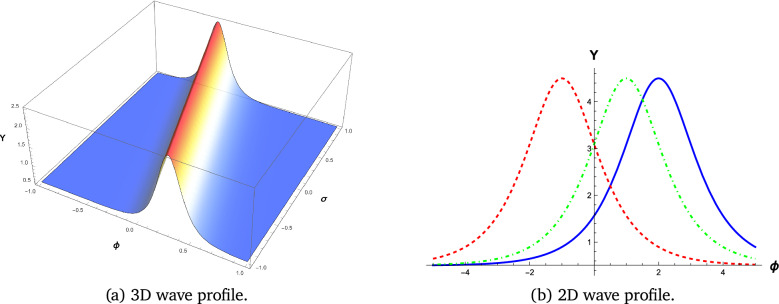
Wave Profile Visualization of equation (7) with parameters $k_1 = 2$, $b_1 = 10$, $b_2 = 5$, $b_3 = -10$, and $c = 3$.Figures generated using Mathematica Version 13.3.1.0 (Wolfram Research, Champaign, IL, USA; https://www.wolfram.com/mathematica/).

**Fig. 3:**
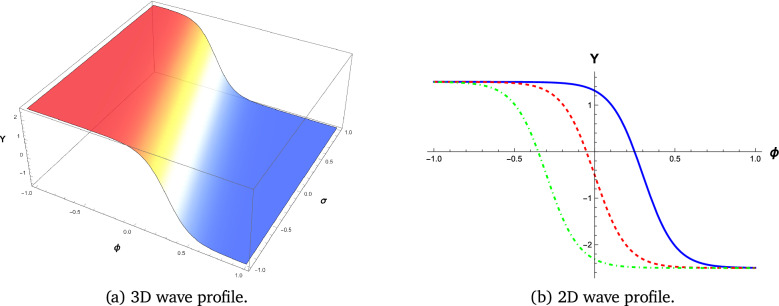
Wave Profile Visualization of equation (8) with parameters $k_1 = 2$, $b_1 = 10$, $b_2 = 5$, $b_3 = -10$, and $c = 3$.Figures generated using Mathematica Version 13.3.1.0 (Wolfram Research, Champaign, IL, USA; https://www.wolfram.com/mathematica/).

**Fig. 4:**
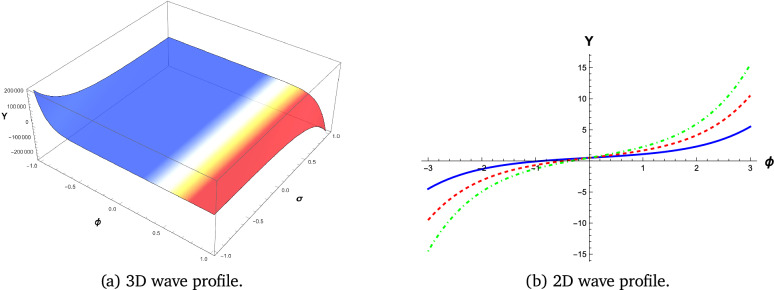
Wave Profile Visualization of equation (9) with parameters $k_1 = 2$, $b_1 = 10$, $b_2 = 5$, $b_3 = -10$, and $c = 3$.Figures generated using Mathematica Version 13.3.1.0 (Wolfram Research, Champaign, IL, USA; https://www.wolfram.com/mathematica/).

We employ visual representations for the novel soliton structures of the non-linear Gardner-KP equation . These pictures show the newly calculated waveform solutions in the form of graphs. The solutions include hyperbolic, trigonometric, and rational forms from different Familys. The physical interpretation of these solutions is facilitated by simulations conducted using the symbolic computation software Mathematica. Soliton structures that can be recognized include double periodic waves, shock wave solutions, kink-shaped solitons, solitary or bell-shaped solitons, and periodic wave soliton solutions. Each has its set of physical properties. The shock wave soliton solution is shown in Fig. 1, the bell-shaped soliton solution is shown in Fig. 2, and the kink type, which shows the smooth transition of soliton solutions, is shown in Figs. 3 and 4.

To provide physical insight, these various soliton solutions correspond to real nonlinear wave phe-nomena observed in fluids and plasma. For example, shock wave solutions model abrupt changes in wave amplitude analogous to physical shock fronts, while bell-shaped solitary waves represent stable waves as they can propagate without distortion and are localized. Kink solitons describe smooth transitions with changing state of waves and are akin to stable domains found in many media. Thus, these solutions outline both valuable mathematical patterns and important meanings related to oceanography, plasma physics and nonlinear wave propagation.

#### Shock wave profile

We evaluate the features of the shock wave solutions described by equation (6). As seen in Fig. 1, the wave is shaped in a certain manner for these selected parameter values. The extreme change in the gradient illustrates the common nonlinear steepening seen in shock effects that happen in fluids and plasmas.

#### Bell-shaped soliton

The wave solution in the form of a bell-shaped soliton appears in Fig. 2. Such a profile remains fixed in shape as it travels, which is common with nonlinear waves, like internal waves found in fluids.

#### Kink-type soliton

Smooth changes from one wave state to another are seen in Figs. 3 and 4 from the kink-type soliton solutions. They show how stable wave motions, like a front, frequently appear in various physical situations.

### Numerical stability analysis of the obtained soliton solutions

For stability, a small amplitude-scaled Gaussian perturbation is added to the above-plotted soliton profile, and these perturbations are observed over time. The perturbation magnitude was evaluated by calculating the norm of the difference between the perturbed and original soliton solutions at various time instances. Every soliton solution showed a decrease in perturbation norm, as the table presented in Table 1 shows, indicating that each soliton is locally stable to small perturbations. Hence, it can be said that solitons are stable when focused on their immediate surrounding points in the solution space^30,31^.

The local stability means that soliton solutions are being considered very close to their exact values. It does not rule out that other types of dynamics and even chaotic dynamics can develop at the larger scales discussed further in the dynamical section.

**Table 1:** Perturbation norms $\Vert \Delta U_i\Vert _2$ of the plotted soliton solutions at selected times.

Time $t$	$\Vert \Delta U_1\Vert _2$	$\Vert \Delta U_2\Vert _2$	$\Vert \Delta U_3\Vert _2$	$\Vert \Delta U_4\Vert _2$
0.00	0.495	0.561	0.384	2.699
1.01	0.299	0.339	0.232	1.629
2.02	0.180	0.204	0.140	0.983
3.03	0.109	0.123	0.084	0.593
4.04	0.066	0.074	0.051	0.358
5.05	0.040	0.045	0.031	0.216
6.06	0.024	0.027	0.019	0.130
7.07	0.014	0.016	0.011	0.079
8.08	0.009	0.010	0.007	0.047
9.09	0.005	0.006	0.004	0.029

## Dynamical analysis of Gardner-Kp equation

Studying the dynamics of the (3+1)-dimensional Gardner-KP equation helps explain the stability of solitons in real situations. Phase portraits, Lyapunov exponents, and Poincaré maps explain how the waves behave, staying the same or sometimes changing to chaos or complex patterns. Seeing these dynamics clearly helps explain how nonlinear waves change, connect, and travel in materials such as fluids and plasma. For such an analysis, we integrate Equation (3) twice to get

$$\Upsilon '' - \frac{2 }{\nu ^2 b_1^2}\Upsilon ^3 + \frac{3}{\nu ^2 b_1^2}\Upsilon ^2+\frac{\mu b_3^2 + c b_1 + \mu b_2^2 }{\nu ^2 b_1^4} \Upsilon = 0.$$

Letting $\alpha _1 = \frac{2 }{\nu ^2 b_1^2}$, $\alpha _2 = \frac{3 }{\nu ^2 b_1^2}$ and $\alpha _3 = \frac{\mu b_3^2 + c b_1 + \mu b_2^2 }{\nu ^2 b_1^4}$ we obtain

$$\Upsilon '' - \alpha _1\Upsilon ^3 + \alpha _2 \Upsilon ^2 + \alpha _3 \Upsilon = 0 .$$

Finally, we apply the Galilean transformation to get

10 $$\begin{aligned} \begin{aligned} {\Upsilon '} = {\Gamma },\ \ \ {\Gamma '} = \alpha _1 {\Upsilon }^3 - \alpha _2 {\Upsilon }^2 - \alpha _3 {\Upsilon }, \end{aligned} \end{aligned}$$

The equilibrium points of (10) are determined by letting

$$\Upsilon ' = 0 \quad \text {and} \quad \Gamma ' = 0.$$

These conditions, when substituted in (10) yield:

$$\Gamma = 0 \quad \text {and} \quad \alpha _1 \Upsilon ^3 - \alpha _2 \Upsilon ^2 - \alpha _3 \Upsilon = 0,$$

which on solving, results in

$$\Gamma = 0 \quad \text {and} \quad \Upsilon = 0, \quad \frac{\alpha _2 \pm \sqrt{\alpha _2^2 + 4\alpha _1\alpha _3}}{2\alpha _1}.$$

The system has three equilibrium points: $E_0(\Upsilon _0, 0)$, $E_1(\Upsilon _1, 0)$, and $E_2(\Upsilon _2, 0)$, where

$$\Upsilon _0 = 0, \quad \Upsilon _1 = \frac{\alpha _2 + \sqrt{\alpha _2^2 + 4\alpha _1 \alpha _3}}{2\alpha _1}, \quad \Upsilon _2 = \frac{\alpha _2 - \sqrt{\alpha _2^2 + 4\alpha _1 \alpha _3}}{2\alpha _1}.$$

The Jacobian matrix for the system (10) can be expressed as

$$J_m(\Upsilon _i, \Gamma _i) = \begin{pmatrix} 0 & 1 \\ 3\alpha _1\Upsilon _i^2 - 2\alpha _2\Upsilon _i - \alpha _3 & 0 \end{pmatrix}, \ i=0, 1, 2.$$

Let $M(\Upsilon _i, 0)$ be the coefficient matrix of the linearized system at the equilibrium point $(\Upsilon _i, 0)$:

$$M(\Upsilon _i, 0) = \begin{pmatrix} 0 & 1 \\ 3\alpha _1\Upsilon _i^2 - 2\alpha _2\Upsilon _i - \alpha _3 & 0 \end{pmatrix} .$$

At the equilibrium point $( \Upsilon _i, 0)$, we define the determinant $J$ and trace $T$ of matrix $M$. The determinant $J$ is given by:

$$J = \alpha _3 + 2\alpha _2\Upsilon -3\alpha _1\Upsilon ^2 ,$$

and the trace $T = 0$.

The eigenvalues of the matrix $M$ are determined by solving the characteristic equation:

$$\begin{aligned} |M - \lambda I_{2 \times 2}| = 0, \end{aligned}$$

which simplifies to

$$\lambda ^2 -(3\alpha _1 \Upsilon _i - 2\alpha _2 \Upsilon _i - \alpha _3)= 0.$$

Thus, the eigenvalues of $M$ are:

$$\lambda _{1,2} = \pm \sqrt{3\alpha _1 \Upsilon _i^2 - 2\alpha _2 \Upsilon _i - \alpha _3} .$$

These eigenvalues depend on the parameters $\alpha _1$, $\alpha _2$, and $\alpha _3$, as well as the equilibrium points $(\Upsilon _i, 0)$. It is important to note that the parameters $\alpha _1$, $\alpha _2$, and $\alpha _3$ are related to system parameters, such as $\nu$, $b_1$, $b_2$, $b_3$, $c$, and $\mu$.

The stability of the critical points $(\Upsilon _i, 0)$ can be analyzed based on Table 2.

As the system (10) is a three-parameter planar dynamical system, with the stability of the system depending on the values of parameters $\alpha _1, \alpha _2,$ and $\alpha _3$. The behavior of the system is further investigated by analyzing the bifurcations in the phase portraits of (10) as the parameter values change.

**Table 2:** Stability classification of equilibrium points based on the Jacobian matrix.

Condition	Eigenvalue Nature	Equilibrium Type	Stability
$J < 0$	Real, opposite signs	Saddle Point	Always unstable
$J > 0$, $T^2 - 4J \ge 0$	Real, same sign	Node	Stable if $T < 0$, unstable if $T > 0$
$J > 0$, $T^2 - 4J < 0$, $T \ne 0$	Complex conjugates	Focus	Stable if $T < 0$, unstable if $T > 0$
$J > 0$, $T = 0$	Pure imaginary	Center	Neutrally stable (closed orbits)
$J = 0$, Poincaré index = 0	Degenerate	Zero Point / Cusp	Indeterminate

### Phase portraits

Phase portraits are graphical representations of a dynamical system’s trajectories in its phase space, showing how the system evolves over time based on initial conditions. They provide insight into the stability and behavior of equilibria, periodic orbits, and chaotic dynamics .

**Fig. 5:**
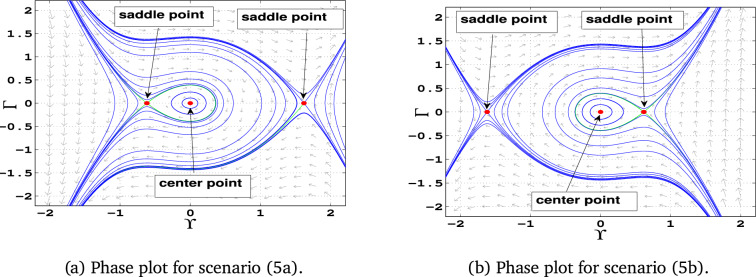
Graphical representations of the system’s phase space dynamics (10).

**Fig. 6:**
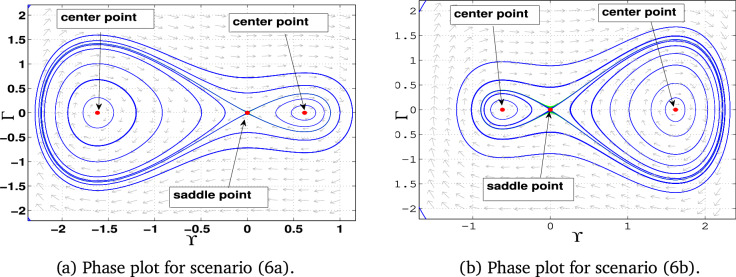
Graphical representations of the system’s phase space dynamics (10).

**Fig. 7:**
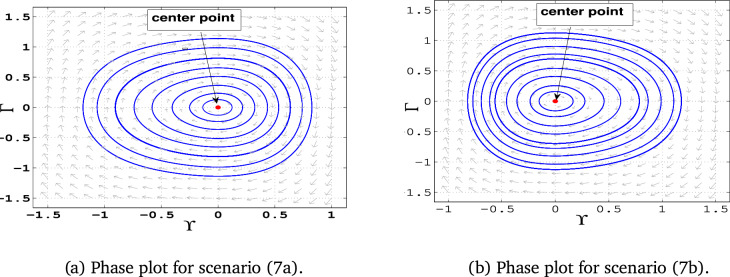
Graphical representations of the system’s phase space dynamics (10).

**Fig. 8:**
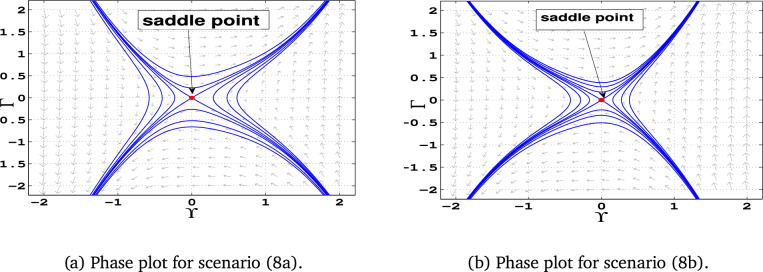
Graphical representations of the system’s phase space dynamics (10).

- **Case 1:** When $\alpha _1$, $\alpha _2$, and $\alpha _3$ are all positive, the system has three equilibrium points: $E_0(0,0)$ and $E_{1\pm }$. The point $E_0$ acts as a center, while the points $E_{1-}$ and $E_{1+}$ are identified as saddle points (Fig. 5-a).
- **Case 2:** In this case, $\alpha _1$ is positive, $\alpha _2$ is negative , $\alpha _3$ is also positive. The system exhibits equilibrium points $E_0(0,0)$ and $E_{2\pm }$. Here, $E_0$ is a center, while $E_{2-}$ and $E_{2+}$ act as saddle points. (Fig. 5-b)
- **Case 3:** If, $\alpha _1$ is negative, $\alpha _2$ is positive , $\alpha _3$ is negative, the system has equilibrium points $E_3(0, 0)$ and $E_{3\pm }$. The equilibrium $E_0$ becomes a saddle point, while $E_{3+}$ and $E_{3-}$ are centers (Fig. 6-a).
- **Case 4:** If all parameters are negative ($\alpha _1, \alpha _2, \alpha _3 < 0$), the system contains equilibrium points $E_0(0, 0)$ and $E_{4\pm }$. In this configuration, $E_0$ is a saddle point, while $E_{4-}$ and $E_{4+}$ are centers (Fig. 6-b).
- **Case 5:** If, $\alpha _1$ is negative, $\alpha _2$ is positive , $\alpha _3$ is also Positive , only one equilibrium point: $E_0(0,0)$, which acts as a center (Fig. 7-a)
- **Case 6:** If, $\alpha _1$ is negative, $\alpha _2$ is negative , $\alpha _3$ is also Positive , the system has only $E_0(0,0)$ as an equilibrium point. This point is identified as a center (Fig. 7-b)
- **Case 7:** If, $\alpha _1$ is positive, $\alpha _2$ is positive , $\alpha _3$ is negative, the system has only $E_0(0,0)$ as an equilibrium point. This equilibrium point functions as a saddle (Fig. 8-a).
- **Case 8:** Finally when, $\alpha _1$ is positive, $\alpha _2$ is negative , $\alpha _3$ is negative the system again has only $E_0(0,0)$ as an equilibrium point. Here, $E_0$ is classified as a saddle (Fig. 8-b).

**Table 3:** Phase Portraits classifications for different values of $\alpha _1$, $\alpha _2$, and $\alpha _3$.

For selecting $\alpha _1$, $\alpha _2$, and $\alpha _3$	Equilibrium points	Eigenvalues	Classifications
$\alpha _1 = 1$, $\alpha _2 = 1$, $\alpha _3 = 1$ Figure 5(a)	(-0.62, 0)	±1.18	Unstable saddle
	(0, 0)	$\pm i$	Stable center
	(1.62, 0)	±1.9	Unstable saddle
$\alpha _1 = 1$, $\alpha _2 = -1$, $\alpha _3 = 1$ Figure 5(b)	(-1.62, 0)	±1.9	Unstable saddle
	(0, 0)	$\pm i$	Stable center
	(0.62, 0)	±1.18	Unstable saddle
$\alpha _1 = -1$, $\alpha _2 = 1$, $\alpha _3 = -1$ Figure 6(a)	(-1.62, 0)	±1.9i	Stable center
	(0, 0)	±1	Unstable saddle
	(0.62, 0)	±1.18i	Stable center
$\alpha _1 = -1$, $\alpha _2 = -1$, $\alpha _3 = -1$ Figure 6(b)	(-0.62, 0)	±1.18i	Stable center
	(0, 0)	±1	Unstable saddle
	(1.62, 0)	±1.9i	Stable center
$\alpha _1 = -1$, $\alpha _2 = 1$, $\alpha _3 = 1$ Figure 7(a)	(0, 0)	$\pm i$	Stable center
$\alpha _1 = -1$, $\alpha _2 = -1$, $\alpha _3 = 1$ Figure 7(b)	(0, 0)	$\pm i$	Stable center
$\alpha _1 = 1$, $\alpha _2 = 1$, $\alpha _3 = -1$ Figure 8(a)	(0, 0)	±1	Unstable saddle
$\alpha _1 = 1$, $\alpha _2 = -1$, $\alpha _3 = -1$ Figure 8(b)	(0, 0)	±1	Unstable saddle

### Hamiltonian dynamics

In classical mechanics, Hamilton’s equations describe systems of the form:

$$\frac{d\Upsilon }{d\chi } = U(\Upsilon , \Gamma ), \quad \frac{d\Gamma }{d\chi } = V(\Upsilon , \Gamma ),$$

A system is Hamiltonian if a function $H(\Upsilon , \Gamma )$ exists such that:

$$U = \frac{\partial H}{\partial \Gamma }, \quad V = -\frac{\partial H}{\partial \Upsilon }.$$

This function $H(\Upsilon , \Gamma )$ is referred to as the Hamiltonian^32^.

#### Definition 1

A system is Hamiltonian if it satisfies:

$$\frac{\partial U}{\partial \Upsilon } + \frac{\partial V}{\partial \Gamma } = 0.$$

Equations describing the system qualify as Hamiltonian if they meet this condition:

$$\frac{\partial }{\partial \Upsilon }\left( \frac{d\Upsilon }{d\chi }\right) + \frac{\partial }{\partial \Gamma }\left( \frac{d\Gamma }{d\chi }\right) = 0.$$

The corresponding Hamiltonian function is:

$$H(\Upsilon , \Gamma ) = \frac{\Gamma ^2}{2} - \frac{\alpha _1}{4} \Upsilon ^4 + \frac{\alpha _2}{3} \Upsilon ^3 + \frac{\alpha _3}{2} \Upsilon ^2.$$

#### Definition 2

For a critical point $(\Upsilon _0, \Gamma _0)$, the discriminant is:

$$\Omega (\Upsilon , \Gamma ) = H_{\Upsilon \Upsilon } \cdot H_{\Gamma \Gamma } - (H_{\Upsilon \Gamma })^2.$$

**Case 1:** When the determinant $\Omega (\Upsilon _0, \Gamma _0)$ is positive, the critical point may be identified as either a local maximum or a local minimum.

**Case 2:** When the determinant $\Omega (\Upsilon _0, \Gamma _0)$ is negative, the critical point corresponds to a saddle point.

**Case 3:** If $\Omega (\Upsilon _0, \Gamma _0) = 0$, further analysis is needed.

**Table 4:** Equilibrium Point Classifications of the System (10) Under Different Parameter Choices for $\alpha _1$, $\alpha _2$, and $\alpha _3$

For selecting $\alpha _1$, $\alpha _2$, and $\alpha _3$	Equilibrium points (Ei, 0)	$\Omega$(Ei, 0)	Classifications
$\alpha _1 = 1, \alpha _2 = 1, \alpha _3 = 1$	(-0.62, 0)	-1.39	Saddle-node
	(0, 0)	1	Stable center
	(1.62, 0)	-3.63	Saddle-node
$\alpha _1 = 1, \alpha _2 = -1, \alpha _3 = 1$	(-1.62, 0)	-3.63	Saddle-node
	(0, 0)	1	Center-node
	(0.62, 0)	-1.39	Saddle-node
$\alpha _1 = -1, \alpha _2 = 1, \alpha _3 = -1$	(-1.62, 0)	3.63	Center-node
	(0, 0)	-1	Saddle-node
	(0.62, 0)	1.39	Center-node
$\alpha _1 = -1, \alpha _2 = -1, \alpha _3 = -1$	(-0.62, 0)	1.39	Center-node
	(0, 0)	-1	Saddle-node
	(1.62, 0)	3.63	Center-node
$\alpha _1 = -1, \alpha _2 = 1, \alpha _3 = 1$	(0, 0)	1	Center-node
$\alpha _1 = -1, \alpha _2 = -1, \alpha _3 = 1$	(0, 0)	1	Center-node
$\alpha _1 = 1, \alpha _2 = 1, \alpha _3 = -1$	(0, 0)	-3.63	Saddle-node
$\alpha _1 = 1, \alpha _2 = -1, \alpha _3 = -1$	(0, 0)	-1	Saddle-node

### Proposed results from Table (3) and Table (4)

**Result 1:** For $\alpha _1 > 0$, $\alpha _3 > 0$, and $\alpha _2^2 + 4\alpha _1\alpha _3 > 0$, the system defined by equation (10) has stable center at $(0, 0)$ and saddle points at:

$$\left( \frac{\alpha _2 + \sqrt{\alpha _2^2 + 4\alpha _1 \alpha _3}}{2\alpha _1}, 0 \right) \quad \text {and} \quad \left( \frac{\alpha _2 - \sqrt{\alpha _2^2 + 4\alpha _1\alpha _3}}{2\alpha _1}, 0 \right)$$

These points represent different dynamical behaviors. Additionally, periodic orbits are observed around the center, and a homoclinic orbit exists at the origin.

**Result 2:** When $\alpha _1< 0$, $\alpha _3 < 0$, and $\alpha _2^2 + 4\alpha _1\alpha _3 > 0$, the system has one saddle point at $(0, 0)$ and two centers located at:

$$\left( \frac{\alpha _2 + \sqrt{\alpha _2^2 + 4\alpha _1 \alpha _3}}{2\alpha _1}, 0 \right) \quad \text {and} \quad \left( \frac{\alpha _2 - \sqrt{\alpha _2^2 + 4\alpha _1\alpha _3}}{2\alpha _1}, 0 \right)$$

This configuration results in a homoclinic orbit around the origin, as well as a family of periodic orbits. These structures point to the presence of periodic, solitary, and breaking wave solutions in the system’s dynamics.

**Result 3:** For $\alpha _2^2 + 4\alpha _1\alpha _3 > 0$ and $\alpha _1 \alpha _3 < 0$, the system has a center point at:

$$\left( \frac{\alpha _2 - \sqrt{\alpha _2^2 + 4\alpha _1\alpha _3 }}{2\alpha _1}, 0 \right)$$

This center point is accompanied by two saddle points, one at the origin and another at

$$\left( \frac{\alpha _2 + \sqrt{\alpha _2^2 + 4\alpha _1\alpha _3}}{2\alpha _1}, 0 \right)$$

This configuration leads to a series of periodic orbits near the center, alongside a homoclinic orbit at the origin. The system exhibits a variety of periodic, solitary, and breaking wave solutions.

**Result 4:** When $\alpha _2^2 + 4\alpha _1 \alpha _3 < 0$, the system has a single equilibrium point at (0,0). This point is saddle if $\alpha _3 < 0$, and a center if $\alpha _3 > 0$. The system also shows a series of bounded open orbits, supporting periodic and breaking wave solutions.

**Result 5:** When $\alpha _2^2 + 4\alpha _1\alpha _3 = 0$, the system defined by equation (10) has one center point at $(0, 0)$ and one saddle point at

$$\left( \frac{\alpha _2}{2\alpha _1}, 0 \right) .$$

### Wave solutions of the dynamical system

**Fig. 9:**
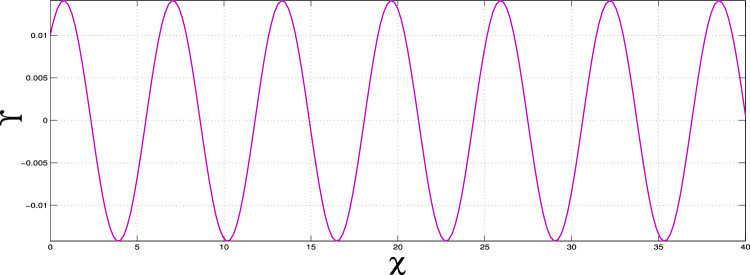
Nonlinear periodic wave solutions of dynamical system (10) for $\alpha _1$, $\alpha _2$, $\alpha _3$ > 0.

**Fig. 10:**
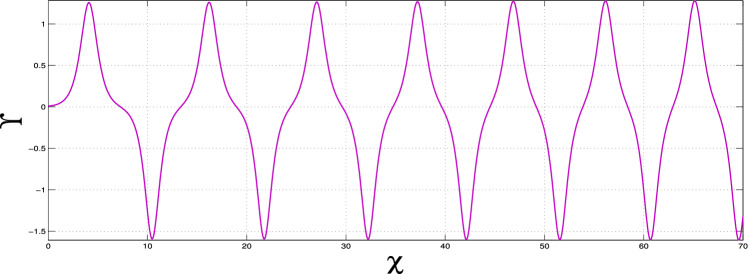
Supernonlinear periodic wave solutions of dynamical system (10) for $\alpha _1=-1$, $\alpha _2=-1$ and $\alpha _3=1$.

To find all possible super-nonlinear wave solutions, we need to figure out all the super-nonlinear paths for system (10) by changing the physical parameters $\alpha _1$, $\alpha _2$, and $\alpha _3$. By systematically adjusting parameters, we illustrate both periodic and super-periodic wave solutions. These results, shown in Figs. 9 and 10, clearly demonstrate that the system can support different types of nonlinear wave behaviors.

Our results clearly show that there are nonlinear periodic wave solutions, as seen in Fig. 9, which shows waves that repeat over time due to nonlinear effects in the system. Additionally, super-nonlinear periodic wave solutions, shown in Fig. 10, are a more complicated type of solution that features stronger nonlinear interactions and more complex wave shapes than regular nonlinear periodic waves. These findings point out the many aspects and levels of detail within the system under study, giving profound insight into the formation of super-nonlinear waves.

## Analysis of quasi-periodic behaviour

In this section, we study the quasiperiodic patterns of the considered model. We explore the quasi-periodic dynamics of the system under three distinct parameter sets, visualizing the results using 2D and 3D phase portraits alongside corresponding time series plots. These visualizations provide a complete view of how the system’s behavior is controlled by parameter change, with subtle interactions between more than one frequency and their impact on stability and predictability.

The perturbed form of Eq (10) after inserting the periodic term $A_0\cos (\tau t)$ is:

11 $$\begin{aligned} \Upsilon ' = \Gamma , \quad \Gamma ' = \alpha _1\Upsilon ^3 - \alpha _2\Upsilon ^2 - \alpha _3\Upsilon + A_0\cos (\tau t), \end{aligned}$$

where $A_0$ is the amplitude and $\tau$ is the frequency of the external forcing.

**Fig. 11:**
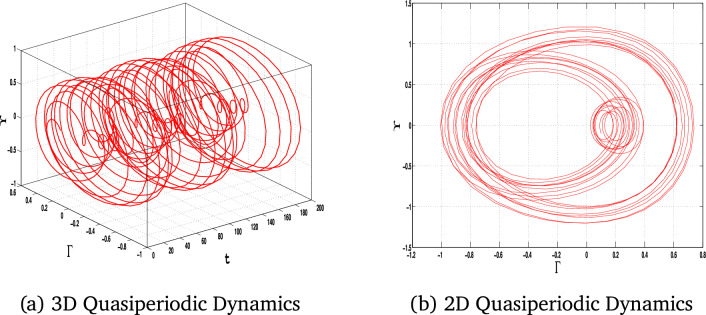
The 3D and 2D phase portraits of the system (11) for $\alpha _1=-1$, $\alpha _2=1$, $\alpha _3=1$ $A_0=0.6$ and $\tau =1.6$.

**Fig. 12:**
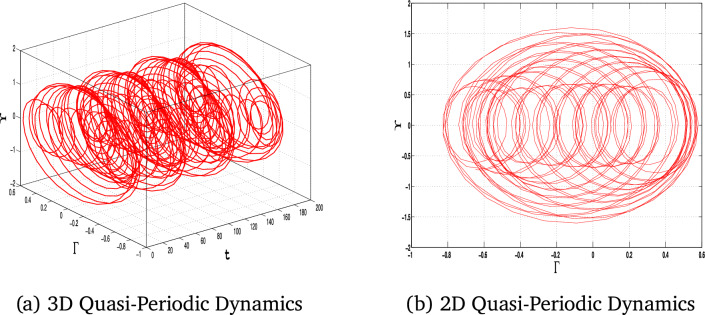
The 3D and 2D phase portraits of the system (11) for for $\alpha _1=-1$, $\alpha _2=1$, $\alpha _3=1$ $A_0=2.5$ and $\tau =3$.

**Fig. 13:**
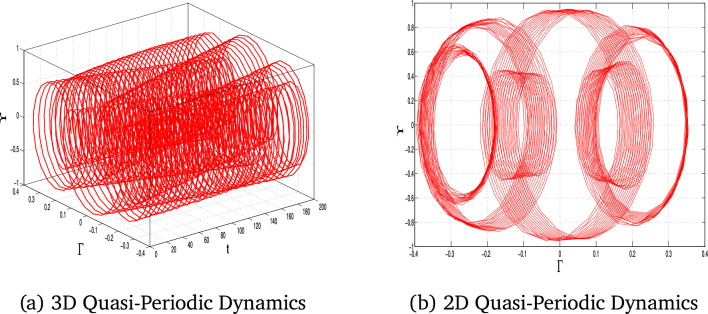
The 3D and 2D phase portraits of the system (11) for for $\alpha _1=-1$, $\alpha _2=1$, $\alpha _3=1$ $A_0=4$ and $\tau =6$.

The results shown in Figs. 11, 12, and 13 for each set of parameters have the defining characteristics of quasi-periodic behavior: bounded but non-repeating orbits for the 2D and 3D plots. This analysis highlights the system’s sensitivity to parameter changes and captures the intricate, deterministic patterns that govern its behavior. These results are necessary to advance our understanding of nonlinear dynamical systems and their potential for applications in real systems. The quasi-periodic patterns that were noted highlight the intricate dynamics of the system, opening the way for a detailed study of chaos through Lyapunov exponent analysis.

### Quantitative insights into dynamical system behavior using Lyapunov exponents

**Fig. 14:**
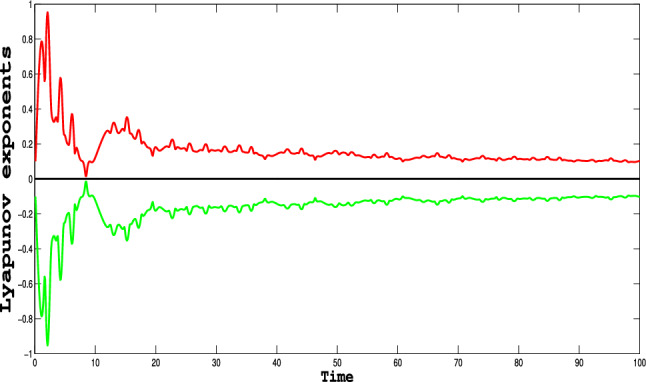
Dynamics of the Lyapunov exponent for the model (11) for the parameters values of $\alpha _1 = -1$,$\alpha _2 = -1$,$\alpha _3 = -1$ , $A_0= 0.2$ and $\tau =0.86$.

Lyapunov exponents are the fundamental quantities that characterize the stability and chaos of dynamical systems. A positive Lyapunov exponent signals exponential divergence of close trajectories, suggesting chaos, while a negative one suggests convergence to a stable state. The results in Table 5 show how the Lyapunov exponents change over time for the system being studied, and Fig. 14 also supports its chaotic behavior with the highest positive Lyapunov exponent of 0.9533. This indication highlights the sensitivity of the system to changes in parameters and initial conditions, giving quantitative verification of the occurrence of chaos. These findings are significant in comprehending the inherent dynamics of nonlinear systems and their potential applications in optimization and control.

**Table 5:** Temporal Variation of Lyapunov Exponents.

Time	Lyapunov Exp1	Lyapunov Exp2
0.1000	0.1008	$-$0.1008
0.2000	0.9533	$-$0.9533
0.5000	0.2450	$-$0.2460
10.0000	0.1235	$-$0.1235
15.0000	0.3231	$-$0.3231
20.0000	0.1815	$-$0.1815
30.0000	0.1625	$-$0.1625
40.0000	0.1420	$-$0.1420
50.0000	0.1549	$-$0.1548
60.0000	0.1156	$-$0.1156
80.0000	0.1140	$-$0.1140
100.0000	0.1028	$-$0.1028

### Poincaré analysis for detecting quasi-periodic and chaotic behaviors

**Fig. 15:**
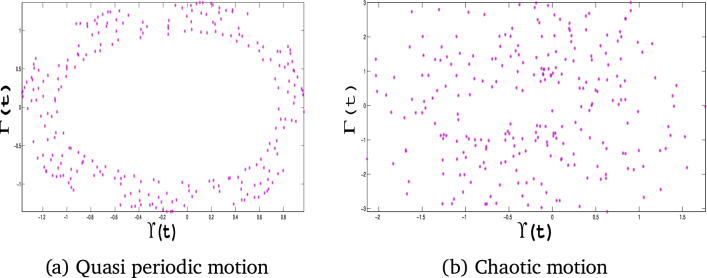
Poincaré maps for the dynamical system (11).

The Poincaré map is a powerful mathematical and visualization technique for analyzing nonlinear dynamical systems. We demonstrate its usefulness in this paper by its capability to distinguish between quasi-periodic and chaotic behavior. Figure 15-a shows a quasi-periodic motion with $\alpha _1 = -1$, $\alpha _2 = 1$, $\alpha _3 = 1$, $A_0 = 0.09$, and $\tau = 1.2$, where the points form a closed and repeating pattern in the Poincaré map. This pattern indicates regularity and stability in the system. In contrast, Fig. 15-b shows chaotic movement when $\alpha _1 = -1$, $\alpha _2 = 1$, $\alpha _3 = 1$, $A_0 = 0.8$, and $\tau = 1.2$, where the scattered points show randomness and how small changes at the start can lead to different outcomes. The results highlight the role of Poincaré maps in identifying complex system behavior and transitions. The results demonstrate how the system reacts to changes in parameters and confirm that this technique is helpful for studying nonlinear dynamics, such as in improving and managing industrial processes.

###  Sensitivity analysis

**Fig. 16:**
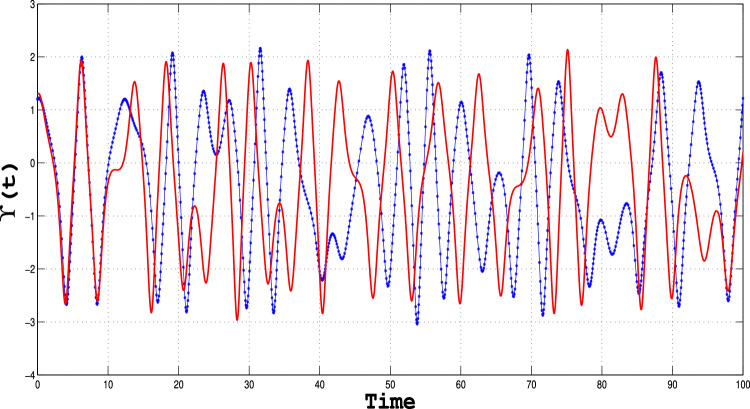
Sensitivity plot for the perturbed dynamical system given in equation ((11)), using the specified parameters and initial conditions outlined in the table.

**Fig. 17:**
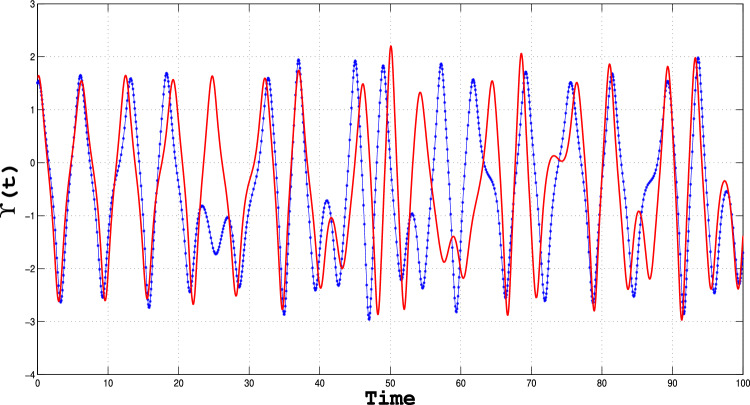
Sensitivity plot for the perturbed dynamical system given in equation ((11)), using the specified parameters and initial conditions outlined in the table.

**Fig. 18:**
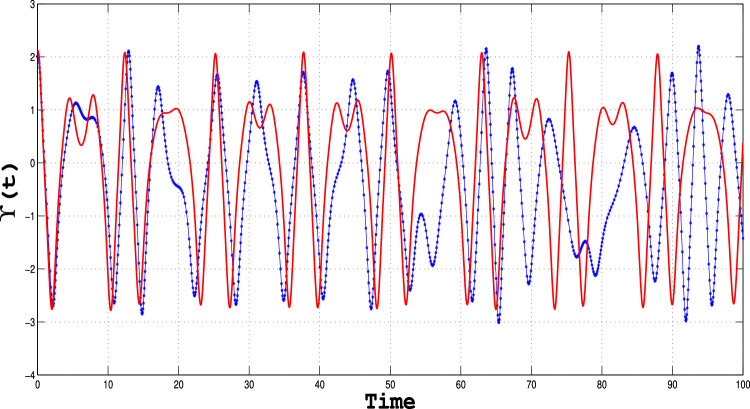
Sensitivity plot for the perturbed dynamical system given in equation ((11)), using the specified parameters and initial conditions outlined in the table.

Sensitivity analysis is the powerful technique that enables one to examine how changes in the input or model parameters alter the output of a system; this provides critical insights into its behavior^33,34^. The results depicted in Figs. 16, 17 and 18) clearly illustrate the system’s sensitivity to initial conditions, confirming the presence of chaotic behavior. These graphs highlight the fundamental aspect of chaos—extreme sensitivity to initial conditions—observed for specific parameter values.

Using the initial conditions outlined in Table 6, we systematically examine how perturbations influence the behavior of the perturbed dynamical system ((11)). The findings underscore the intricate interplay between system parameters and initial states, demonstrating the critical role of sensitivity analysis in uncovering the rich dynamics of chaotic systems^28^.

**Table 6:** Parametric values for sensitivity analysis.

Dynamical system Type	Initial conditions data		
	Figure	Blue Curve	Red Curve
System ((11))	16	(1.2,0.2)	(1.3, 0.3)
System ((11))	17	(1.5,0.5)	(1.6,0.6)
System ((11))	18	(2,0.6)	(2.1,0.6)

## Interpretation of results and applications

The results from this research have greatly contributed to increasing knowledge on Gardner-KP type equations. Most previous studies have dealt with (2+1)-dimensional Gardner-KP and related equations^20–22,25,26^, mainly investigating simple soliton patterns such as single solitons, lumps and breathers. On the other hand, this study on the (3+1)-dimensional Gardner-KP equation reveals various soliton profiles, like double periodic waves, shock waves and kink-type solitons.

A thorough study of the system here discovers new kinds of stability and leads to chaotic behavior that past studies have rarely described. It is evident from this that (3+1)-dimensional models are better for reflecting the various aspects of nonlinear waves in physical systems.

Multiple fields could make use of the outcomes from this work.

- Ocean Engineering plays a role by studying how internal waves travel along the ocean’s shelves and impact movement of sediments and shaping of coasts.
- Exploring wave distributions in plasma which cover solitary pulses and shock waves important for energy processes.
- how solitons behave in optical fibers, necessary for securing the digital signal of communication systems.
- Bose-Einstein Condensates with their self-organizing nature help us study nonlinear waves and their stability in quantum fluids.

Overall, this study broadens the application of Gardner-KP models in several scientific fields by adding concepts and explanations for practical use.

## Conclusion

We investigated the non-linear $(3+1)$-dimensional KP-Gardner equation, focusing on its soliton solutions and dynamical behaviors. Using the Jacobi elliptic method, we derived soliton solutions that illustrate the equation’s rich nonlinear wave dynamics. These solitons provide valuable insights into wave transmission dynamics, with applications in various fields of physics and engineering. We conducted the dynamical analysis using the phase plane analysis, which provided a detailed classification of phase portraits based on orbital structures. Sensitivity analysis results were presented to highlight the system’s dependence on parameter variations. We used the Runge-Kutta (RK) method to solve the model, which indicated the existence of both supernonlinear and nonlinear periodic wave patterns. We further explored the effects of physical constants on quasi-periodic and chaotic patterns within the perturbed dynamical system. To confirm chaotic behavior, Lyapunov exponents were calculated, Poincaré sections were plotted, and sensitivity analysis was performed. Changes in the frequencies and strengths of outside disturbances greatly affect the system’s unpredictable, chaotic behavior, as these analyses revealed. The results have profound implications for applications in engineering, fiber optics, and other scientific disciplines, where nonlinear models are critical to advancing theoretical and practical innovations.

## Data Availability

The datasets used and/or analysed during the current study available from the corresponding author on reasonable request.
